# The bench scientist's guide to statistical analysis of RNA-Seq data

**DOI:** 10.1186/1756-0500-5-506

**Published:** 2012-09-14

**Authors:** Craig R Yendrek, Elizabeth A Ainsworth, Jyothi Thimmapuram

**Affiliations:** 1 USDA ARS Global Change and Photosynthesis Research Unit, 1201 W. Gregory Drive, Urbana, IL 61801, USA; 2 Department of Plant Biology, University of Illinois, Urbana-Champaign, Urbana, IL, 61801, USA; 3 Roy J. Carver Biotechnology Center, University of Illinois, Urbana-Champaign, Urbana, IL, 61801, USA; 4 Current Address: Bioinformatics Core, Discovery Park, Purdue University, West Lafayette, IN, 47907, USA

**Keywords:** RNA-Seq, Differential Expression, Statistical analysis

## Abstract

**Background:**

RNA sequencing (RNA-Seq) is emerging as a highly accurate method to quantify transcript abundance. However, analyses of the large data sets obtained by sequencing the entire transcriptome of organisms have generally been performed by bioinformatics specialists. Here we provide a step-by-step guide and outline a strategy using currently available statistical tools that results in a conservative list of differentially expressed genes. We also discuss potential sources of error in RNA-Seq analysis that could alter interpretation of global changes in gene expression.

**Findings:**

When comparing statistical tools, the negative binomial distribution-based methods, edgeR and DESeq, respectively identified 11,995 and 11,317 differentially expressed genes from an RNA-seq dataset generated from soybean leaf tissue grown in elevated O_3_. However, the number of genes in common between these two methods was only 10,535, resulting in 2,242 genes determined to be differentially expressed by only one method. Upon analysis of the non-significant genes, several limitations of these analytic tools were revealed, including evidence for overly stringent parameters for determining statistical significance of differentially expressed genes as well as increased type II error for high abundance transcripts.

**Conclusions:**

Because of the high variability between methods for determining differential expression of RNA-Seq data, we suggest using several bioinformatics tools, as outlined here, to ensure that a conservative list of differentially expressed genes is obtained. We also conclude that despite these analytical limitations, RNA-Seq provides highly accurate transcript abundance quantification that is comparable to qRT-PCR.

## Findings

### Background

As a method for characterizing global changes in transcription, RNA-Seq is an attractive option because of the ability to quantify differences in mRNA abundance in response to various treatments and diseases, as well as to detect alternative splice variants and novel transcripts [1]. Compared to microarray techniques, RNA-Seq eliminates the need for prior species-specific sequence information and overcomes the limitation of detecting low abundance transcripts. In addition, early studies have demonstrated that RNA-Seq is very reliable in terms of technical reproducibility [2]. As a result, biologists studying an array of model and non-model organisms are beginning to utilize RNA-Seq analysis with ever growing frequency [3-7]. However, without experience using bioinformatics methods, the large number of choices available to analyze differential expression can be overwhelming for the bench scientist (see Table one in [8]).

Essentially, RNA-Seq consists of five distinct phases, 1) RNA isolation, 2) library preparation, 3) sequencing-by-synthesis, 4) mapping of raw reads to a reference transcriptome or genome and 5) determining significance for differential gene expression (for review see [1]). In an effort to familiarize the bench scientist with the post-sequencing analysis of RNA-Seq data (phase 5), we have developed an analysis strategy based on currently available bioinformatics tools. Here, we compare three statistical tools used to analyze differential gene expression: edgeR, DESeq and Limma [9-11]. Based on their performance, we present an analysis strategy that combines these tools in order to generate an optimized list of genes that are differentially expressed. Finally, we highlight several aspects of RNA-Seq analysis that have the potential to lead to misleading conclusions and discuss options to minimize these pitfalls.

### Results

#### Generating high quality reads is dependent on initial RNA quality

Prior to library construction and sequencing-by-synthesis, the quality of the isolated RNA was assessed by gel electrophoresis to ensure purity (Additional file 1). Three replicate samples were isolated from soybean leaves that had been grown in either chronic O_3_ (150 parts per billion) or ambient O_3_ for six weeks. No degradation was observed in any of the samples and staining of the 26S rRNA band was more intense compared to the 18S rRNA band, indicating that high quality RNA had been isolated. In addition, there was no evidence that genomic DNA was co-purified during RNA extraction. Following library preparation and sequencing-by-synthesis, analysis of the raw reads determined that all six samples had a median quality score (QS) of 34 (Table 1). As a result, averages of ~28 million high quality reads were obtained for each sample.

**Table 1:** Post sequencing analysis of raw reads

**Sample**	**Treatment**	**Flowcell lane**	**Number of reads**	**Q.S. (median)**	**Q.S. (interquartile range)**
1	Ambient	4	36,408,402	34	26-36
2	Elevated O_3_	4	28,554,551	34	26-36
3	Ambient	5	16,862,414	34	29-37
4	Elevated O_3_	5	17,575,844	34	29-37
5	Ambient	6	31,889,531	34	28-37
6	Elevated O_3_	6	37,605,167	34	28-37

For each sample, the total number of reads and read quality score (QS) is listed. A QS of 34 indicates one sequencing error per 4,000 base pairs. Generally, a QS over 20 (1% error rate) is considered acceptable for RNA-Seq. One control (−) and one elevated ozone (+) replicate were pooled and run on a single lane of the flow-cell.

#### Utilizing statistical tools that are compatible with RNA-Seq data

The raw reads described in Table 1 were aligned to the soybean reference transcriptome [12] using the mapping tool Novoalign, a short read aligner demonstrated to be highly accurate [13,14]. When differential expression was analysed subsequently, the total number of genes with significantly altered transcript abundance in plants exposed to elevated ozone was 11,995 for edgeR, 11,317 for DESeq and 9,131 for Limma. Since RNA-Seq generates count data, it is more appropriate to use a discrete probability distribution to analyze differential gene expression [15]. Therefore, edgeR and DESeq, which are based on the negative binomial distribution, are compatible with the data generated by RNA-Seq [9,10]. In contrast, Limma [16] was adapted to analyze RPKM values using a method previously developed for continuous data from microarray studies (fluorescence values) and is based on the *t*-distribution [11]. The Limma method was clearly very different from the two negative binomial distribution methods, but even between edgeR and DEseq there were 678 additional genes identified by edgeR as differentially expressed, representing approximately 6% of the significant genes.

#### Workflow for RNA-Seq data optimization

**Figure 1:**
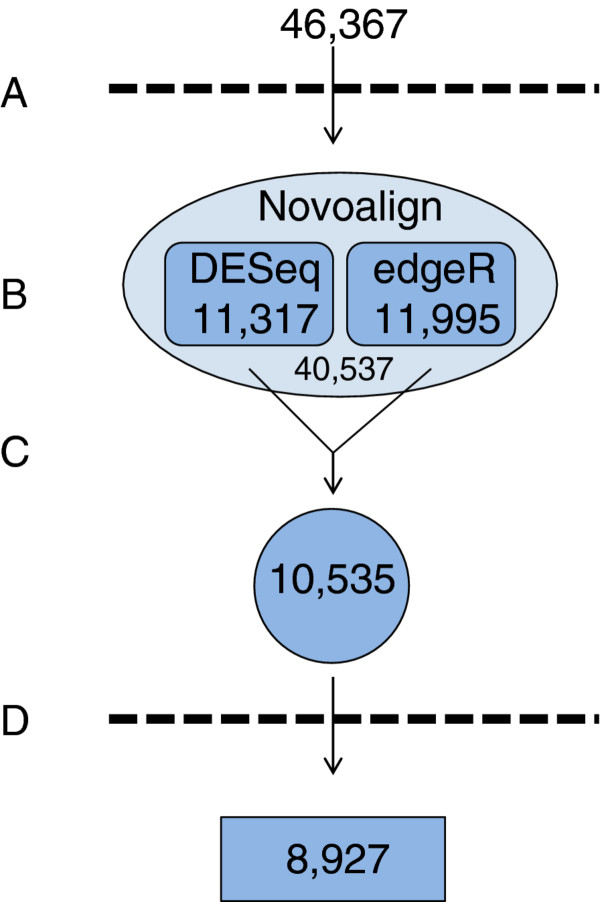
**RNA-Seq data optimization strategy.** The flowchart outlines the strategy for identifying soybean leaf transcripts significantly changing in response to elevated ozone. All genes mapping zero reads for all samples were removed (**A**) after aligning raw reads to the reference transcriptome, consisting of 46,367 genes. Differential expression was then separately determined using DESeq and edgeR (**B**). The two lists of significant genes were intersected to obtain a single list of differentially expressed genes (**C**). Finally, low expression genes (RPKM < 1.0) were removed (**D**).

In response to the differences described above, we developed a strategy to integrate the results analyzed separately by edgeR and DESeq into one optimized dataset. As a first step, any gene that had zero mapped reads for all six samples was removed, resulting in 40,537 genes mapped by Novoalign out of the 46,367 genes comprising the soybean reference transcriptome (Figure 1, Step A) [12]. Software code to carry out this preliminary step as well as the subsequent analyses using edgeR and DESeq (Figure 1, Step B) using the R statistical package [17] is provided (Additional file 2). These analyses are performed independently using the same mapping file (Additional file 3) and result in two excel files containing log_2_ fold change values and *p*-values that have been adjusted for multiple testing for each gene that was mapped by Novoalign.

**Figure 2:**
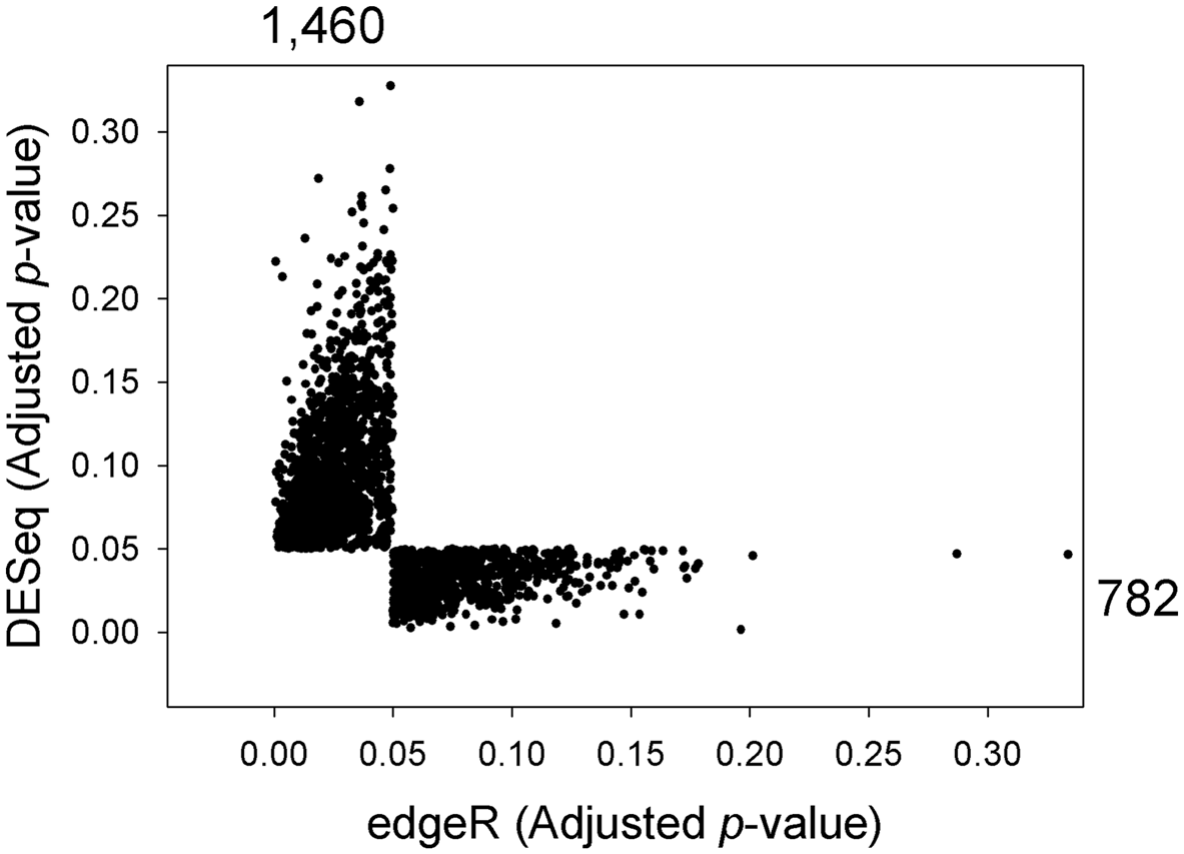
***p*-value comparison between edgeR and DESeq.** The edgeR and DESeq *p*-values of the 2,242 marginally significant genes eliminated in Step C of Figure 1 are compared.

In order to identify the common genes determined to be differentially expressed by both DESeq and edgeR, we intersected the two lists of significant genes (Figure 1, Step C). As a result, the genes that were determined to be significantly regulated by only one statistical method were eliminated. A comparison of the 2,242 eliminated genes revealed that the non-significant *p*-value responsible for the gene's removal was generally close to, but above *p* = 0.05 (Figure 2). Therefore, we classified these genes as marginally significant. The optimized list after these filtering and merge steps totalled 10,535 differentially expressed genes. Many of these genes had very low read counts for all samples, potentially making conclusions related to biological relevance misleading. To deal with this issue, we removed any gene with a control and treatment RPKM value of < 1.0 (Figure 1, Step D), reducing the total number of differentially expressed genes to 8,927. However, this step is optional and should be performed only after careful consideration.

#### Comparing the accuracy of RNA-seq data with qRT-PCR

**Figure 3:**
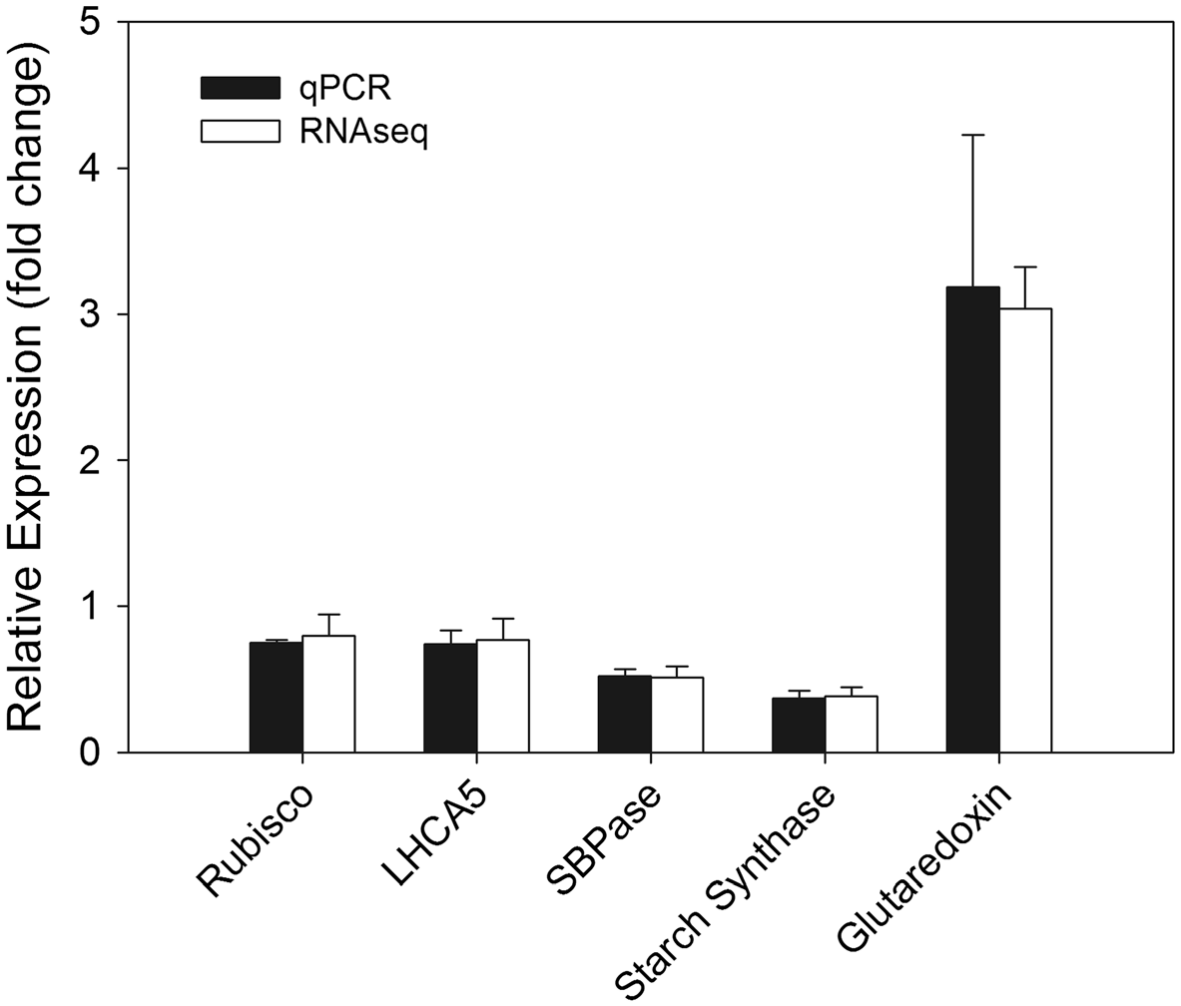
**Comparing the accuracy of RNA-Seq data using qRT-PCR.** Relative expression ratios determined by qRT-PCR were compared to RNAseq results for several genes known to be regulated by elevated ozone.

Several genes known to be regulated by elevated ozone were chosen to analyze via qRT-PCR. The targets chosen include genes involved with photosynthesis, carbohydrate metabolism and oxidative stress, all biological processes that have been well characterized to be responsive to elevated ozone at the level of transcription [18]. The response of each of the targets was consistent with the documented effects of elevated ozone. In addition, the expression ratios for both methods were similar (Figure 3), thus validating the previously reported accuracy of RNA-Seq data.

#### Potential pitfalls and limitations of RNA-Seq analysis

A first potential limitation of this approach is that it may be too conservative, as evidenced by the 2,242 marginally significant genes that were removed from the final optimized list (Figure 1, Step C). The behavior of these genes was analysed in the context of changes to transcripts with broadly similar functions, using the MapMan expression tool [19] to analyze functional category significance for each of the lists of marginally significant genes (Table 2). This tool first identified 11 functional categories from the optimized list of differentially expressed genes consisting of a subset of genes that collectively responded to elevated ozone in a similar manner; i.e., the expression profile of each significant functional category was different from the expression profile of all other categories. When the lists of marginally significant genes were analyzed subsequently, most of these categories were found not to be significantly different, indicating that the eliminated genes did not respond in a manner similar to the optimized list of genes. However, statistical significance was achieved for several categories. Despite having an expression profile consistent with the remaining genes included in the optimized list, 320 RNA, 70 stress, 36 hormone metabolism, 19 DNA, and 10 mitochondrial electron transport-related genes were eliminated based on a non-significant determination by one of the two statistical tools.

**Table 2:** Functional category significance of optimized and marginally significant genes

**Functional Category**	**Optimized**		**DESeq marginal**		**edgeR marginal**	
	**# of genes**	***p*-value**	**# of genes**	***p*-value**	**# of genes**	***p*-value**
Stress	497	0 *	70	2.20E-03 *	19	0.17
Signaling	909	0 *	102	0.43	40	0.70
Cell wall	263	8.51E-29 *	28	0.14	4	0.50
Photosynthesis	117	3.79E-05 *	22	0.76	4	0.23
RNA	1132	6.04E-05 *	222	0.01 *	98	4.40E-03 *
Hormone metabolism	321	3.08E-04 *	36	0.01 *	19	0.51
DNA	133	0.002 *	34	0.70	19	0.03 *
Major CHO metabolism	76	0.003 *	5	0.72	7	0.42
Lipid metabolism	223	0.023 *	23	0.34	16	0.88
Mitochondrial electron transport / ATP synthesis	71	0.042 *	2	0.17	10	0.04 *
TCA cycle	44	0.049 *	---	---	7	0.53

The genes eliminated from Step C in Figure 1 are grouped into functional categories and compared with the final optimized list of significant genes. *p*-value indicates the significance that transcript abundance of all the genes within a specified category are changing in a similar manner compared to all other categories. Asterisks signify *p*-value below p = 0.05.

**Figure 4:**
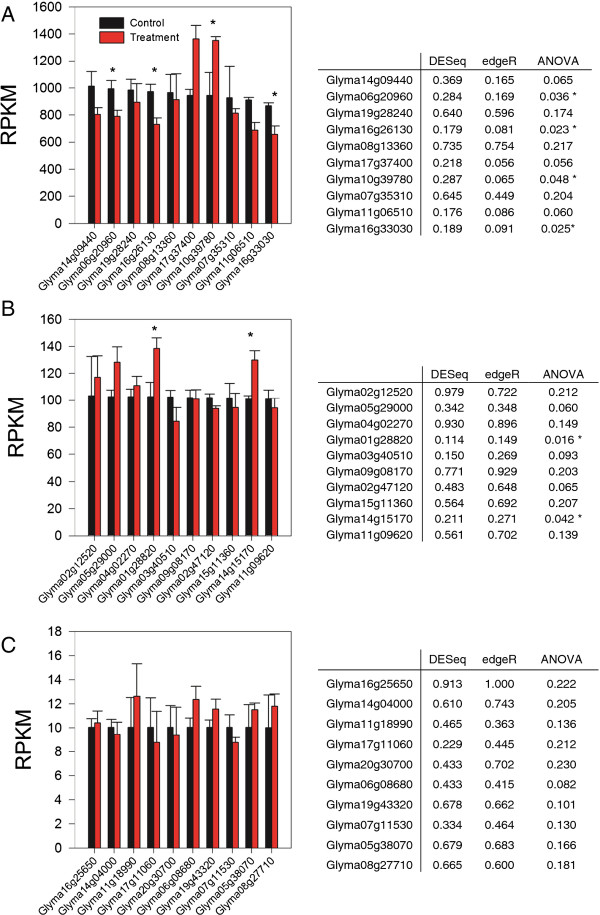
**Identification of type II error across a range of transcript abundance levels.** RPKM values were compared between control and treatment for 10 randomly selected genes, ranging from high (**A**), moderate (**B**) and low (**C**) abundance transcripts. Also included are the *p*-values from DESeq, edgeR and an ANOVA performed using RPKM data. Asterisks signify *p*-value below p = 0.05.

An additional limitation was uncovered by further investigation of the final list of optimized genes. After a cursory examination of several genes that were previously characterized to be regulated by growth in elevated ozone, we identified a potential issue with the statistical analysis that preferentially impacted the high abundance genes. It is well-documented that plants grown in elevated ozone exhibit reduced photosynthesis, increased antioxidant capacity and increased protein turnover [18]. Four high abundance genes (Glyma05g25810, Glyma20g27950, Glyma17g37280 and Glyma11g11460) involved with these processes were not found to be differentially expressed by at least one of the statistical tools used in this analysis, despite RPKM values with obvious differences and analysis of variance (ANOVA) results that indicated significance (Table 3). A more detailed examination across a range of RPKM values support the finding of an increase in type II error for high abundance genes. Four out of 10 randomly selected genes with RPKM values near 1000 that were determined not to be differentially regulated by both edgeR and DESeq did, in fact, have significantly altered transcript abundance when analyzed using ANOVA (Figure 4A). In contrast, none of the genes with RPKM values near 10 were identified as false negatives (Figure 4C).

**Table 3:** Statistical limitations are revealed by independent analysis of ozone-responsive genes

**Functional annotation**	**Locus ID**	**Transcript length**	**Control RPKM**	**Treatment RPKM**	**Fold change**	**DESeq**	**edgeR**	**Individual *t*-test**
Light-harvesting complex II CAB protein	Glyma05g25810	1100	7733.20 ± 783.0	4575.86 ± 429.3	0.59	0.063	3.39E-04 *	0.004 *
Ubiquitin	Glyma20g27950	1540	1504.18 ± 149.8	2425.15 ± 206.1	1.61	0.061	0.007 *	0.003 *
Thioredoxin	Glyma17g37280	1134	265.49 ± 11.1	220.79 ± 17.8	0.83	0.14	0.213	0.021 *
L-ascorbate peroxidase	Glyma11g11460	1278	84.73 ± 2.6	107.98 ± 4.4	1.27	0.214	0.272	0.001 *
Polysaccharide catabolism	Glyma06g45700	1831	70.47 ± 8.6	18.91 ± 9.2	0.27	2.34E-29 *	4.48E-19 *	0.002 *
Glutaredoxin	Glyma13g30770	747	11.31 ± 1.4	34.68 ± 6.8	3.07	8.39E-14 *	1.56E-11 *	0.004 *
Protein degradation DER1 like	Glyma04g14250	1088	5.23 ± 0.2	44.52 ± 11.8	8.51	7.06E-49 *	8.30E-38 *	0.005 *
Lipoxygenase	Glyma03g42500	2833	2.90 ± 0.4	5.64 ± 1.3	1.95	1.96E-04 *	2.26E-04 *	0.027 *
Starch synthase catalytic domain	Glyma20g36040	1954	2.84 ± 0.8	0.12 ± 0.1	0.04	1.14E-23 *	1.60E-36 *	0.005 *
WRKY trascription factor	Glyma10g27860	1468	1.69 ± 0.4	70.92 ± 20.80	41.97	9.23E-121 *	2.76E-92 *	0.005 *

Genes known to be regulated by elevated ozone that had a range of transcript abundances were selected from the optimized list of differentially regulated genes. In addition to *p*-values from DESeq and edgeR, an ANOVA was performed on RPKM values. Asterisks signify *p*-value below p = 0.05.

### Discussion

While the aim of this paper is to familiarize the molecular biologist interested in undertaking an RNA-Seq project with the methods and issues related to post-sequencing analysis, emphasis still needs to be placed on proper handling of RNA samples. Here, we isolated high quality RNA (Additional file 1) using a well-established protocol for soybean leaf tissue [20]. In addition, care was taken during the library construction and sequencing-by-synthesis phases, as evidenced by the high quality scores for each sample (Table 1). As a result, the average number of usable reads per sample was >20 million, which is the recommended depth required to quantify differential expression in a species with a referenced genome [21].

It is also important to utilize a valid experimental design for RNA-Seq projects, which includes the use of biological replicates. Reports demonstrating highly reproducible RNA-Seq results [2,22] make it tempting to reduce sequencing costs by only using one replicate per treatment group. However, without replication it is impossible to estimate error, without which there is no basis for statistical inference [23]. Therefore, it is recommended that RNA-Seq experiments include at least three biological replicates per treatment group [24], as was done in the experiment presented here.

Along these lines, it is important to understand the nature of RNA-Seq data and why it is necessary to use a compatible statistical method, such as a negative binomial distribution [9,10]. For discrete variables such as count data, it is possible to associate all observed values with a non-zero probability. In contrast, there is zero probability that a specific fluorescence value (continuous variable) will be obtained from microarray hybridization. This distinction is important in the context of the varying number of total reads obtained for individual RNA-Seq samples. For example, the probability of mapping 100 reads out of 16.86 million (Table 1; Sample3) for a particular gene is different than mapping 100 reads out of 36.41 million (Table 1; Sample1). To deal with this issue, both edgeR [9] and DESeq [10] normalize the read data based on the total number of reads per sample prior to differential expression analysis.

The main goal of this work was to compare the accuracy of two statistical tools, edgeR and DEseq. At first glance, it appears that both tools perform equally well (Figure 1, Step B). However, when the differentially expressed genes from edgeR and DEseq were intersected (Figure 1, Step C), quite a few genes from each list were eliminated (2,242 total genes). Because of this, we adopted a strategy to identify genes that were determined to be differentially expressed by both edgeR and DESeq. In other words, greater confidence was achieved if a gene was determined significant by each of the statistical tools.

This strategy made it possible to follow the genes that were eliminated and to identify aspects of the analysis that have the potential to lead to erroneous conclusions. One aspect to consider is how each of the different statistical tools is designed to handle and report ‘zero reads’ or transcripts that are not expressed in a given treatment. For example, DESeq will output 'Inf' or '-Inf' to excel as the log_2_ fold change value for genes that fail to align any reads for all control or treatment samples (Table 4). In contrast, edgeR outputs log_2_ fold changes values that are unrealistically large. It is possible that some of these genes could reveal important aspects of global transcription that were altered (i.e., genes that were turned on or off by the treatment) and should not be inadvertently removed. In many cases, however, these genes had very few reads for each replicate as well as for each treatment (Table 4). Transcript abundance this low, while determined to be significantly different, is unlikely to be biologically relevant and should be removed from the analysis. Care should be taken when choosing an arbitrary cutoff, however, to prevent the elimination of genes that may play a transcriptional role in response to the treatment being investigated. In this case, we used a conservative RPKM value <1.0 that resulted in the removal of 1,608 low abundance genes (Figure 1, Step D).

**Table 4:** Expression data for low abundance genes

	**DESeq**		**edgeR**		**Raw Counts**						**RPKM**	
	**log2 FC**	**Padj**	**log2 FC**	**Padj**	**con1**	**con2**	**con3**	**trt1**	**trt2**	**trt3**	**Control**	**Treatment**
***Genes turned on***												
Glyma18g02680	Inf	0.0224	27.39	0.0187	0	0	0	5	1	4	0	0.08 ± 0.032
Glyma01g41980	Inf	0.0331	27.26	0.0187	0	0	0	3	4	2	0	0.30 ± 0.206
Glyma11g04880	Inf	0.0324	27.25	0.0187	0	0	0	5	2	2	0	0.17 ± 0.054
Glyma16g06500	Inf	0.0320	27.24	0.0326	0	0	0	5	1	3	0	0.14 ± 0.057
Glyma12g05780	Inf	0.0488	27.08	0.0326	0	0	0	3	1	4	0	0.06 ± 0.030
***Genes turned off***												
Glyma07g02590	-Inf	0.0011	−28.11	0.0004	7	4	5	0	0	0	0.20 ± 0.050	0
Glyma17g17930	-Inf	0.0011	−28.12	0.0004	3	5	8	0	0	0	0.17 ± 0.084	0
Glyma17g34230	-Inf	0.0016	−28.02	0.0006	9	3	3	0	0	0	0.54 ± 0.292	0
Glyma12g14620	-Inf	0.0052	−27.71	0.0035	3	5	4	0	0	0	0.57 ± 0.372	0
Glyma03g37640	-Inf	0.0075	−27.56	0.0061	4	2	5	0	0	0	0.14 ± 0.013	0

Log_2_ fold change, *p*-value, raw count data and RPKM vaules for representative samples from gene clusters turned on or off by elevated ozone. DESeq outputs an 'Inf' or '-Inf' log2 fold change value to excel when all control or treatment replicates map zero reads.

Another aspect that has the potential to confound RNA-Seq analysis deals with the issue of statistical stringency. In Table 2, we demonstrated that for several functional categories, the marginally significant genes eliminated from the optimized list did, in fact, respond to elevated ozone in a manner similar to the genes ultimately determined to be differentially expressed. Therefore, it may be more appropriate to perform network analysis for individual metabolic or signal transduction pathways using the entire RNA-Seq dataset, not just the genes determined to be differentially expressed [25]. However, this strategy is limited by pathways that have been previously characterized and would fail to uncover new connections, especially unknown signalling relationships.

One final issue revealed by this analysis was the increase in type II error for high abundance genes (Table 3 and Figure 4). Several of the genes determined not to be differentially regulated by one or both of the statistical tools are involved with processes that have been well characterized to be regulated to elevated ozone, including decreased photosynthesis (Glyma05g25810 and Glyma17g37280) [16], increased antioxidant capacity (Glyma11g11460) [26] and increased protein turnover (Glyma20g27950) [27]. However, these genes were determined to be differentially expressed based on statistical analysis of RPKM values. This problem undermines the effectiveness of performing RNA-Seq analysis to uncover novel relationships because it will fail to identify all of the high abundance genes that are differentially regulated in response to elevated ozone; genes that are more likely to impact biological processes, especially metabolic functions.

### Conclusions

There are many new challenges facing the bench scientist when undertaking an RNA-Seq project, especially regarding the large number of bioinformatics tools that have been developed to analyze the post-sequencing dataset [28-32]. Here, we provide a step-by-step guide for analyzing RNA-seq data. In addition, we identified limitations that exist for widely used methods to determine differential expression of RNA-seq data. Therefore, we suggest that our strategy to merge the common genes identified by multiple tools and examine the eliminated genes is an improvement that better ensures confidence in generating a list of differentially expressed genes. We also demonstrate that the results obtained from a select set of genes using qRT-PCR closely agree with the RNA-Seq data. Because of this high accuracy, we envision RNA-Seq replacing microarrays as the new standard for global transcript quantification.

### Methods

#### Background

Soybean plants (*Glycine max* cv. Be Sweet 292) were grown in environmentally controlled growth chambers for six weeks in either ambient or elevated ozone conditions (150 ppb for 8 h d^-1^). Tissue was collected from mature leaves and ground to a fine powder in a liquid nitrogen cooled mortar and pestle. Total RNA was isolated following the protocol of Bilgin et al. [20] and DNase treated using the TURBO DNA-free kit (Life Technologies, Grand Island, NY). Each sample (5 μg) was resolved on a 1% agarose gel containing 40 mM MOPS (pH 7.0), 2 mM EDTA (pH 8.0) and 5 mM iodoacetamide. Before loading the gel, each sample was diluted to 10μL with nuclease free water and heated at 70°C for 5 min along with 7.5μL MOPS/EDTA buffer and 5μL formaldehyde (37% wt.).

#### Library preparation and sequencing-by-synthesis

The DNase-treated RNA (1 μg) was used to prepare individually barcoded RNA-Seq libraries with the TruSeq RNA Sample Prep kit (Illumina, San Diego, CA). Pools of two samples per lane were sequenced on a HiSeq2000 for 100 cycles using version 2 chemistry and analysis pipeline 1.7 according to the manufacturer's protocols (Illumina, San Diego, CA). All raw data has been submitted to the NCBI [GenBank:SRP009826].

#### Aligning raw reads to the soybean transcriptome

Illumina sequences from each of the samples from three biological replicates of control and treatment (elevated ozone) were cleaned using the FASTX toolkit, with a minimum quality score of 20 and minimum length of 75 nt. Soybean genome (Gmax_109) and gff file (Gmax_109.gff3) were downloaded from phytozome ( http://www.phytozome.net/soybean). Soybean transcripts were extracted from the genome sequences based on the.gff file. These soybean transcripts (46,367 transcripts) were considered as reference transcriptome for RNA-Seq analysis.

Mapping of Illumina sequences with Novoalign was done with –H (for hard clipping the reads), –l 65, -rA10 (to allow 10 multiple alignments). With these parameters at least 90% of the each read's length should map to the reference to consider it as a mapped read. After mapping with Novoalign, read counts for each gene were generated using PERL scripts. These reads counts were used for statistical analysis using DESeq and edgeR packages of ‘R’ to determine differential expression at the gene level. Since approximately 92% of the mapped reads aligned to the transcriptome uniquely, multireads were not considered. All biological replicates demonstrated a >0.93 correlation when RPKM values were compared, indicating high reproducibility of replicates. See online user guides for more information about performing alignments with Novoalign ( http://www.novocraft.com/wiki/tiki-index.php).

#### Statistical analyses

Gene lengths and count data for the three independent control and ozone-treated replicates were used to analyze differential expression using R software (Version 2.13.0) [33]. The Limma-RPKM method is based on a two-group Affymetrix dataset design included as part of the Limma package [11,17]. For the edgeR analysis, the trimmed mean of the M values method (TMM; where M = log_2_ fold change) was used to calculate the normalization factor and quantile-adjusted conditional maximum likelihood (qCML) method for estimating dispersions was used to calculate expression differences using an exact test with a negative binomial distribution [9,15,34]. For the DESeq analysis, differential expression testing was performed using the negative binomial test on variance estimated and size factor normalized data [10]. All *p*-values presented were adjusted for false discovery rate to control for type I error due to multiple hypothesis testing. The programming code for each of the specific packages can be found by viewing the vignette details in R using the 'openVignette()' command.

Log_2_ fold change values were loaded into the MapMan expression tool to link gene identifiers with functional annotations using the Gmax_109_peptide mapping file. This tool automatically analyzes functional category significance base on the Wilcoxon rank sum test [19].

Differential expression of RPKM normalized data was tested by ANOVO and corrected for multiple comparisons following the methods of Benjamini and Hochberg (1995) [35] with a false discovery rate of 0.25 using SAS (Version 9.2, Cary, NC; Table 4).

#### qRT-PCR

First-strand cDNA synthesis was performed using 1 μg of DNase treated RNA and was reverse transcribed in a 20 μl reaction with Superscript II (Life Technologies, Grand Island, NY) and oligo(dT) primers according to the manufacturer's instructions. Quantitative PCR was performed on an Applied Biosystems 7900HT Fast Real-Time PCR System (Life Technologies, Grand Island, NY) using Power SYBR Green PCR master mix (Life Technologies, Grand Island, NY) and 400nM of each primer in a 10 μl reaction. Primers were aliquoted onto a 384-well PCR plate using a JANUS automated liquid handling system (Perkin Elmer, Waltham, MA). The following are the primer sequences for each of the target genes: Rubisco (Glyma19g06340), primer A- GCACAATTGGCAAAGGAAGT, primer B- GAGAAGCATCAGTGCAACCA; LHCA5 (Glyma06g04280), primer A- GTGGAGCATCTTTCCAATCC, primer B- TGGATAAGCTCAAGCCCAAG; SBPase (Glyma11g34900), primer A- ATAAGTTGACCGGCATCACC, primer B- GGGTTGTCAGATGTGGCTCT; starch synthase (Glyma13g27480), primer A- GACCCTCTCGATGTTCAAGC, primer B- ATTCTCTGAGGTGGCAATGG; glutaredoxin (Glyma13g30770), primer A- AATCCAATGGCACCTATCCA, primer B- AGGGTTCACTCCCAGACCTT. Target gene expression was normalized to cons14 [36]. Each PCR amplification curve was analyzed with LinRegPCR software [37] to calculate the PCR efficiency and threshold value from the baseline-corrected delta-Rn values in the log-linear phase. The normalized expression level for each gene was determined as reported in [38].

## Availability of supporting data

The data set supporting the results of this article is included within the article (and its additional files).

## Competing interests

The authors declare that they have no competing interests.

## Authors’ contributions

CRY and EAA conceived of the initial experiments. CRY grew the soybean plants, isolated the RNA, developed the analysis strategy, carried out the analysis, and prepared the manuscript. JT performed the mapping of raw reads to the reference transcriptome and performed the initial RPKM, DESeq and edgeR analyses. All authors were involved with discussion regarding the experimental design and approved the final manuscript.

## Supplementary Material

Additional file 1 **RNA quality assessment.** Five μg of total RNA for each sample was run on a 1% agarose gel. See Table 1 for description of sample number treatment. Click here for file

Additional file 2 **R software code.** R software code used to analyze differential expression of the mapping file using edgeR and DESeq. Click here for file

Additional file 3 **Mapping file.** Excel file consisting of raw reads mapped to the soybean reference transcriptome. Click here for file
