# Supplementary figures and images for: The bench scientist's guide to statistical analysis of RNA-Seq data
Source: BMC Res Notes. 2012 Sep 14;5:506. doi: 10.1186/1756-0500-5-506 (PMC3522531; doi:10.1186/1756-0500-5-506)

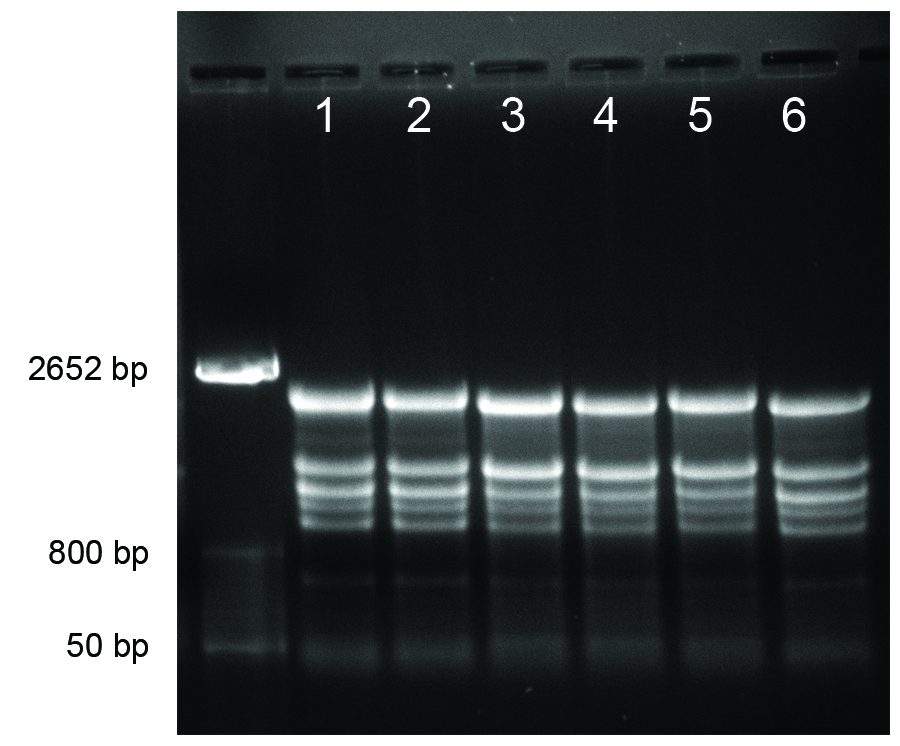

Supplement: Additional file 1 — RNA quality assessment. Five μg of total RNA for each sample was run on a 1% agarose gel. See Table 1 for description of sample number treatment. [file 1756-0500-5-506-S1.tiff]
