# Supplementary material for: The bench scientist's guide to statistical analysis of RNA-Seq data
Source: BMC Res Notes. 2012 Sep 14;5:506. doi: 10.1186/1756-0500-5-506 (PMC3522531; doi:10.1186/1756-0500-5-506)
Supplement: Additional file 2 — R software code. R software code used to analyze differential expression of the mapping file using edgeR and DESeq. [file 1756-0500-5-506-S2.pdf]

R codes for RNA-Seq analysis. User defined descriptions are required in green text.

Load raw reads and remove genes with zero reads for all samples.

```
setwd("C:/Set the working directory to the one where the data is located")
options(help_type="text")
options(stringsAsFactors = FALSE)
library(affycoretools)
library(limma)
library(edgeR)
library(DESeq)
raw.data <- read.delim("Novoalign_transcriptome.txt",stringsAsFactors=F)
targets <-
data.frame(FileName=colnames(raw.data)[3:8],Group=rep(c("trt","con"),each=3))
targets$Gp2 <- factor(targets$Group)
targets$Gp2 <- as.numeric(targets$Gp2)
plotDensity(log2(raw.data[,3:8]+0.1), xlab="log2 raw counts")
library.sizes <- colSums(raw.data[,3:8])
raw.data$trtTotal <- rowSums(raw.data[,3:5])
raw.data$conTotal <- rowSums(raw.data[,6:8])
raw.data$allTotal <- rowSums(raw.data[,3:8])
raw.data <- raw.data[raw.data$allTotal != 0 ,]
```

Calculate RPKM values

```
rpkm.data <-
matrix(NA,nrow=nrow(raw.data),ncol=6,dimnames=list(raw.data$Gene,targets$FileName))
for (i in 1:6) {
    rpkm.data[,i] <- raw.data[,2+i] / (raw.data$Length/1000) /
(library.sizes[i]/1000000)
    }
write.csv(rpkm.data,file="rpkm_values_Novoalign.csv")
```

Calculate normalization factors for edgeR analysis and perform edgeR analysis

```
rownames(raw.data) <- raw.data$Gene
d <- DGEList(counts=as.matrix(raw.data[,3:8]), lib.size=library.sizes,
            group=targets$Group)
d <- calcNormFactors(d)
d <- estimateCommonDisp(d)
de.com <- exactTest(d,pair=c("con","trt"))
edgeR.detailed <- topTags(de.com,n=Inf)
write.csv(edgeR.detailed$table,file="edgeR_results.csv")
```

Calculate normalization factors for DESeq analysis and perform DESeq analysis.

```
cds <- newCountDataSet(raw.data[,3:8],targets$Group)
cds <- estimateSizeFactors(cds)
cds <- estimateVarianceFunctions( cds )
vsd <- getVarianceStabilizedData( cds )
DESeq.detailed <- nbinomTest( cds, "con", "trt" )
write.csv(DESeq.detailed,file="DESeq_results.csv")
```
